# Early Prediction of Neurological Outcome After Cardiac Arrest‐Rationale and Design of the Prospective International Observational EARLY‐NEURO, a STEPCARE Substudy

**DOI:** 10.1111/aas.70239

**Published:** 2026-04-17

**Authors:** Marion Moseby‐Knappe, Erik Westhall, Marjolein Admiraal, Margareta Lang, Helena Levin, Johanna Hästbacka, Marjaana Tiainen, Markus Skrifvars, Gisela Lilja, Janus C. Jacobsen, Alice Lagebrant, Matt Wise, Matti Reinikainen, Paul Young, Manoj Saxena, Simon Schmidbauer, Naomi Hammond, Frances Bass, Ameldina Ceric, Caroline Kamp, Christina Sillassen, Christoph Leithner, Pascal Stammet, Matthias P. Hilty, Pedro D. Wendel‐Garcia, Georg Royl, Tobias Graf, Matt Thomas, Katie Sweet, Stepani Bendel, Joonas Tirkkonen, Annerose Mengel, Maria‐Ioanna Stefanou, Luis Georg Romundstad, Philipp Seidel, Jessica Kåhlin, Jonathan Grip, Jonna Heinonen, Rakesh H. Jadav, Jens Nee, Daniela Nowak, Matthias Hänggi, Johan Undén, Anna Lybeck, Joachim Düring, Martin Kenda, Jesper Johnsson, Niklas Nielsen, Tobias Cronberg

**Affiliations:** ^1^ Department of Neurological Rehabilitation Skåne University Hospital Lund Sweden; ^2^ Department of Clinical Sciences Lund Lund University Lund Sweden; ^3^ Department of Clinical Neurophysiology Skåne University Hospital Lund Sweden; ^4^ Department of Radiology Helsingborg Hospital Helsingborg Sweden; ^5^ Department of Research, Development, Education and Innovation Skåne University Hospital Lund Sweden; ^6^ Faculty of Medicine and Health Technology, Tampere University Hospital Wellbeing Services County of Pirkanmaa and Tampere University Tampere Finland; ^7^ Department of Neurology Helsinki University Hospital and University of Helsinki Helsinki Finland; ^8^ Department of Anesthesia and Intensive Care Helsinki University Hospital and University of Helsinki Helsinki Finland; ^9^ Department of Neurology Skåne University Hospital Lund Sweden; ^10^ Copenhagen Trial Unit, Centre for Clinical Intervention Research Copenhagen University Hospital‐Rigshospitalet Copenhagen Denmark; ^11^ Department of Regional Health Research, Faculty of Health Sciences University of Southern Denmark Odense Denmark; ^12^ Department of Neurology Skåne University Hospital Malmö Sweden; ^13^ Adult Critical Care University Hospital of Wales Cardiff UK; ^14^ Institute of Clinical Medicine University of Eastern Finland Kuopio Finland; ^15^ Department of Anesthesiology and Intensive Care Kuopio University Hospital Kuopio Finland; ^16^ Intensive Care Unit Wellington Hospital Wellington New Zealand; ^17^ Medical Research Institute of New Zealand Wellington New Zealand; ^18^ Australian and New Zealand Intensive Care Research Centre Monash University Melbourne Victoria Australia; ^19^ Department of Critical Care University of Melbourne Melbourne Victoria Australia; ^20^ Critical Care Division and Department of Intensive Care Medicine, the George Institute for Global Health and St George Hospital Clinical School University of New South Wales Sydney Australia; ^21^ Department of Anesthesia and Intensive Care Skåne University Hospital Malmö Sweden; ^22^ Critical Care Program, the George Institute for Global Health UNSW Sydney Randwick New South Wales Australia; ^23^ Malcolm Fisher Department of Intensive Care Royal North Shore Hospital, Northern Sydney Local Health District St Leonards New South Wales Australia; ^24^ The George Institute for Global Health Sydney Australia; ^25^ Royal North Shore Hospital Sydney Australia; ^26^ Charité‐Universitätsmedizin Berlin, Department of Neurology Freie Universität and Humboldt‐Universität Zu Berlin Berlin Germany; ^27^ Department of Anesthesia and Intensive Care Medicine Centre Hospitalier de Luxembourg Luxembourg Luxembourg; ^28^ Department of Life Sciences and Medicine, Faculty of Science, Technology and Medicine University of Luxembourg Esch‐sur Alzette Luxembourg; ^29^ Institute of Intensive Care Medicine University Hospital Zurich Zurich Switzerland; ^30^ Medical Faculty University of Zurich Zurich Switzerland; ^31^ Division of Cardiothoracic Anaesthesia and Intensive Care Medicine, Department of Anaesthesia, Intensive Care Medicine and Pain Medicine Medical University of Vienna Vienna Austria; ^32^ Department of Neurology University Hospital Schleswig‐Holstein Campus Lübeck Lübeck Germany; ^33^ University Heart Center Lübeck University Hospital Schleswig‐Holstein Schleswig‐Holstein Germany; ^34^ German Center for Cardiovascular Research (DZHK) Hamburg Germany; ^35^ Intensive Care Unit University Hospitals Bristol and Weston NHS Foundation Trust Bristol UK; ^36^ Department of Intensive Care Kuopio University Hospital Kuopio Finland; ^37^ Intensive Care Unit Tampere University Hospital Tampere Finland; ^38^ Department of Neurology and Stroke Eberhard‐Karls University of Tübingen Tübingen Germany; ^39^ Hertie Institute of Clinical Brain Research University Medicine Tübingen Germany; ^40^ Department of Neurology and Stroke University Medicine Tübingen Germany; ^41^ Second Department of Neurology “Attikon” University Hospital, School of Medicine, National and Kapodistrian University of Athens Athens Greece; ^42^ Department of Anesthesia and Intensive Care Medicine, Division of Emergencies and Critical Care Oslo University Hospital Oslo Norway; ^43^ Lovisenberg Diaconal University College Oslo Norway; ^44^ Department of Intensive Care Medicine Stavanger University Hospital Stavanger Norway; ^45^ Department of Quality and Health Technology, Faculty of Health Sciences University of Stavanger Stavanger Norway; ^46^ Perioperative Medicine and Intensive Care Karolinska University Hospital Stockholm Sweden; ^47^ Department of Physiology and Pharmacology Karolinska Institutet Stockholm Sweden; ^48^ Department of Clintec Karolinska Institute Stockholm Sweden; ^49^ Department of Neurophysiology University Hospital of Wales Cardiff UK; ^50^ Department of Nephrology and Intensive Care Medicine Charité—Universitätsmedizin Berlin Berlin Germany; ^51^ Department of Operation and Intensive Care Hallands Hospital Halmstad Sweden; ^52^ Department of Anesthesia and Intensive Care, Clinical Sciences Malmö Lund University Lund Sweden; ^53^ Department of Anesthesia and Intensive Care Skane University Hospital Lund Sweden; ^54^ Department of Clinical Sciences, Helsingborg Lund University Helsingborg Sweden; ^55^ Department of Anesthesia and Intensive Care Medicine Skåne University Hospital Malmö Sweden; ^56^ Department of Neurology University Hospital LMU Munich Munich Germany; ^57^ Department of Anesthesiology and Intensive Care Helsingborg Hospital Helsingborg Sweden

**Keywords:** brain injury markers, cardiac arrest, CT, EEG, outcome, prognostication

## Abstract

**Background:**

Guidelines discourage prediction of neurological outcome in comatose patients within the first 72 h after cardiac arrest. Increasing evidence suggests that patients with the most severe brain injury and those with no or minimal brain injury may be identified before 72 h using novel methods. We present a protocol for the EARLY‐NEURO study, which aims to evaluate whether good and poor outcomes can be reliably predicted already from 24 h after cardiac arrest using the most commonly available methods.

**Methods:**

Protocol for a prospective international multicenter substudy within the Sedation, TEmperature and Pressure after Cardiac Arrest and REsuscitation (STEPCARE) trial where adults post‐arrest are randomized to minimal or deep sedation, fever treatment with or without a temperature management device and to two different targets of mean arterial blood pressure. Patients sedated or still unconscious at 24 h are examined with head computed tomography (CT) and electroencephalogram (EEG). Blood samples are collected at 24 h after randomization, and stored for analysis of the brain injury marker neurofilament light. CT and EEG examinations will be centrally evaluated for signs of a likely poor or good outcome applying standardized criteria by raters blinded to treatment allocations and patient outcomes. Intensive care treatment, neurological prognostication, and criteria for withdrawal of care will be according to the STEPCARE protocol. Timepoint and reasons for withdrawal of life‐sustaining therapy (WLST) will be recorded. WLST prior to 72 h after randomization based on a presumed futile neurological prognosis is strongly discouraged. Primary outcome will be good or poor functional outcome, assessed by the modified Rankin Scale (dichotomized as 0–3 versus 4–6) at 6 months. Results will be reported in accordance with the Standards for Reporting Diagnostic Accuracy (STARD).

**Conclusions:**

Earlier prognostication aims to balance the avoidance of premature treatment withdrawal in patients with favorable potential against the prevention of unnecessary intervention in patients with a definitely poor prognosis.

## 1 Background

The European Resuscitation Council and the European Society of Intensive Care Medicine (ERC/ESICM) recommend a standardized multimodal approach for the prediction of neurological outcome after 72 h in patients unconscious after cardiac arrest to avoid falsely pessimistic predictions [1]. In a recent retrospective analysis combining data from four clinical studies, including more than 4000 international cardiac arrest patients, we found that any two prognostic routine methods including neuroimaging, specific clinical neurological signs, neurophysiological examinations and levels of brain injury markers in blood accurately identified poor outcome patients [2]. We also found that neuroprognostication was unreliable if only one of these prognostic routine methods indicated poor outcome [2]. The ERC/ESICM prognostic algorithm was modified in 2025 to also include criteria of a likely favorable outcome with the aim of reducing the number of patients with an indeterminate neurological prognosis [1, 3, 4, 5].

In recent years, the prognostication of neurological outcome in unconscious cardiac arrest patients has improved: there have been efforts toward standardized evaluations of the most commonly used add‐on examinations electroencephalography (EEG) and computed tomography (CT) [6, 7, 8, 9]. Further, the brain injury marker Neurofilament light (NFL) has demonstrated its superiority in predicting unfavorable outcomes compared to the currently recommended routine marker Neuron‐specific enolase, although optimal cutoff levels remain uncertain [1, 5, 10].

In the early 2000s, induced hypothermia was recommended as a neuroprotective intervention following cardiac arrest. However, owing to insufficient evidence to support induced hypothermia, several guidelines now prioritize normothermic temperature control and fever prevention [4]. This has reduced the need for sedation to that required for comfort and clinical procedures. Subsequently, we hypothesize that the recommended delay of neuroprognostication until 72 h post‐arrest to reduce the confounding by sedatives may not be strictly necessary in all cases [1]. The information used in routine neuroprognostication is often continuously collected up to the time point of 72 h. Several prognostic methods including EEG, CT and biomarkers have demonstrated high prognostic performance for prediction of good and poor functional outcome within the first 24 h after cardiac arrest [11, 12, 13, 14, 15, 16, 17]. Sedation is unlikely to affect the prognostic performance of CT and biomarkers; however, this has not been examined in a randomized controlled trial.

Here we present the rationale and statistical analysis plan for a prospective international multicenter neuroprognostic study, the EARLY‐NEURO, nested within the international randomized clinical Sedation, TEmperature and Pressure after Cardiac Arrest and REsuscitation (STEPCARE) trial [18, 19, 20, 21]. The STEPCARE trial randomizes patients to three parallel interventions related to sedation levels, temperature control, and mean arterial blood pressure.

The aim of this study is to evaluate whether early, multimodal neurological prognostication using a combination of clinical neurological examinations, CT, EEG, and NFL can predict good and poor outcomes in patients unconscious after out‐of‐hospital cardiac arrest of any cause. Together, these methods evaluate structural brain injury, neuronal electric activity, and the quantity of neuronal breakdown products in blood.

The design of the STEPCARE further enables a unique opportunity to study the impact of the level of sedation (deep versus minimal) on the prognostic performances of guideline recommended methods. Two STEPCARE interventions, fever management and blood pressure targets, are not expected to influence the reliability of the prognostic performance, but will also be reported in this study.

**FIGURE 1:**
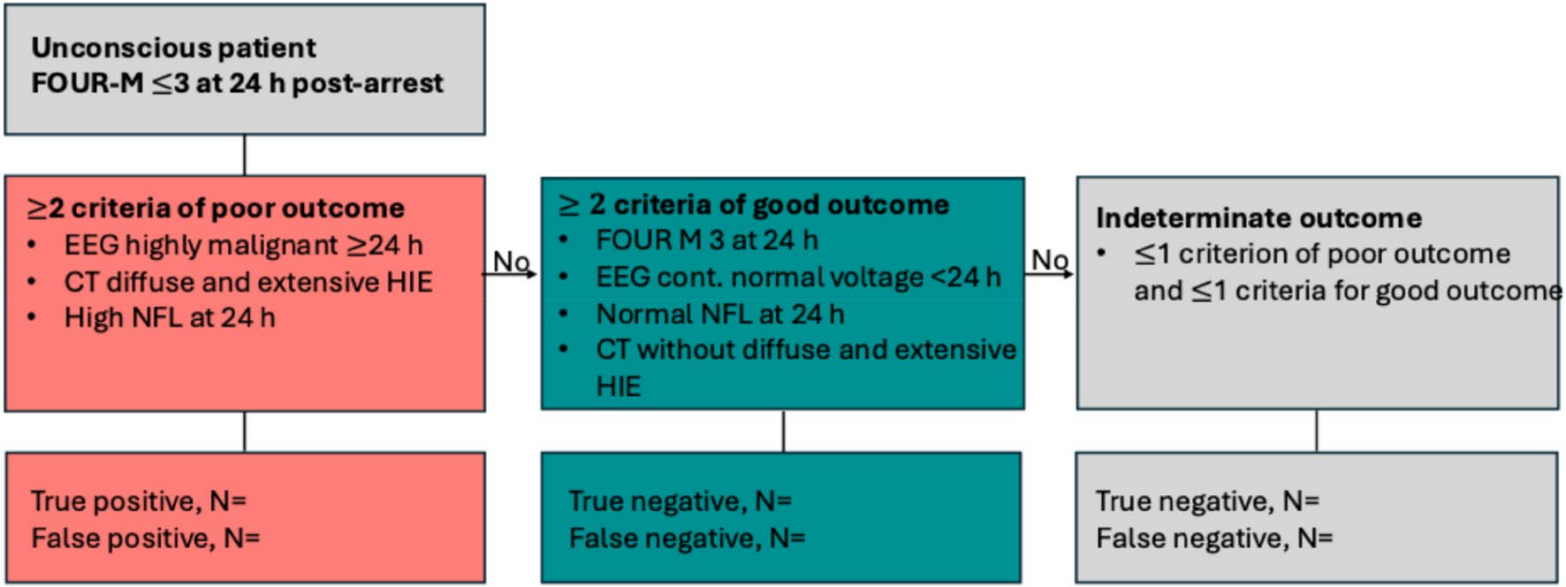
Study hypotheses. Patients unconscious at 24 h after cardiac arrest, defined as not being awake and following commands (FOUR‐M—score ≤ 3) will be evaluated according to the following criteria for likely poor (mRS 4–6) and good (mRS 0–3) outcome at 6 months. Criteria for poor outcome include electroencephalogram (EEG) highly malignant patterns (suppression or burst‐suppression) at ≥24 h, computed tomography (CT) with diffuse and extensive hypoxic‐ischaemic brain injury (HIE) ≥ 6 h after cardiac arrest or high blood levels of neurofilament light (NFL) at 24 h. Criteria for good outcome include localizing a painful stimulus on clinical neurological examination (FOUR‐M = 3), EEG with a continuous normal voltage background < 24 h, normal blood levels of NFL at 24 h or CT without diffuse and extensive HIE. Patients not fulfilling either criteria for likely poor outcome or good outcome likely will remain classified as indeterminate outcome. True positive: pathological criteria for poor outcome and poor functional outcome. False positive: pathological criteria for poor outcome and good functional outcome. True negative: good outcome and either criteria for good outcome or indeterminate outcome. False negative: poor outcome and either criteria for good outcome or indeterminate outcome. For patients with indeterminate outcome, the concordance and discordance between criteria of good and poor outcome prediction and the actual functional outcome will be presented in a separate table as described in the supplementary.

*Our primary hypotheses are*:

Any combination of two of the following criteria predicts poor functional outcome at 6 months without any false positive assessments in patients unconscious (defined as unable to obey verbal commands) at 24 h after cardiac arrest (Figure 1):

- A highly malignant EEG pattern ≥ 24 h after cardiac arrest defined as burst‐suppression or suppression with or without superimposed discharges, defined according to the American Clinical Neurophysiology Society (ACNS) terminology [22].
- Diffuse and extensive hypoxic‐ischaemic brain injury (HIE) on CT according to qualitative evaluation or a gray‐white matter ratio (GWR‐8) < 1.10 at the basal ganglia level [8].
- High blood levels of NFL at 24 h after cardiac arrest defined by the 2% False Positive Rate (FPR) from an ongoing individual data meta‐analysis in adult out‐of‐hospital cardiac arrest patients.

Any combination of 2 of the following criteria predict good functional outcome at 6 months after cardiac arrest with high sensitivity:

- Localizing to a painful stimulus (Full Outline of Unresponsiveness Motor Score (FOUR‐M) 3) on clinical examination at 24 h after cardiac arrest.
- A continuous or nearly continuous and normal voltage > 20 μV EEG background ≤ 24 h after cardiac arrest on continuous EEG‐monitoring, or as soon as possible after 24 h if routine EEG is used.
- Normal NFL blood levels at 24 h after cardiac arrest are defined by the laboratory standard levels.
- Absence of diffuse and extensive HIE on a CT taken as soon as possible after 24 h after admission to intensive care.

*Our secondary hypotheses are*:

- Sedation strategy, fever management with or without a device or blood pressure targets will not have a clinically relevant effect on the prognostic performance of the prognostic examinations above.

*Additional hypotheses that may be reported in separate publications*:

- No patient fulfilling criteria for a poor outcome using any guideline recommended method (Figure 1) will have normal blood levels of NFL. Since patients with rapid brain death may be an exception from this hypothesis, we will present these patients in a subgroup analysis.
- The sensitivity and specificity of the primary hypotheses of the EARLY‐NEURO are not inferior to the sensitivity and specificity of the ERC/ESICM recommended algorithm at 72 h after cardiac arrest.
- Patients fulfilling criteria of brain death will likely demonstrate earlier signs of hypoxic–ischaemic brain injury on CT than other poor outcome patients.
- NFL levels at 24 h will correlate with the neurocognitive outcome measures Montreal Cognitive Assessment (MoCA) and the Symbol Digit Modalities Test (SDMT) at 6 months.
- MIRACLE_2_ (Missed, Initial rhythm, Reactive pupils, Age, Changing rhythm, Low pH, Epinephrine given) risk scores correlate with neurological outcome groups (good, indeterminate, and poor) according to the EARLY‐NEURO main hypotheses.
- The combination of tests to predict poor and good outcome according to the EARLY‐NEURO primary hypothesis perform equally well if NFL is substituted for the routine marker Neuron‐specific enolase (NSE) at 24 h.

## 2 Methods

### 2.1 Study Design

The EARLY‐NEURO is a prospective observational international substudy using the infrastructure of the Sedation, TEmperature and Pressure after Cardiac Arrest and REsuscitation (STEPCARE) trial (Clinicaltrials.gov STEPCARE: NCT05564754, EARLY‐NEURO: NCT05706194). STEPCARE is an investigator‐initiated 2 × 2 × 2 randomized clinical trial randomizing within 4 h after return of spontaneous circulation as outlined below.

### 2.2 Inclusion Criteria

Adult patients ≥ 18 years with an out‐of‐hospital cardiac arrest from any cause are eligible for inclusion in the STEPCARE as described [18, 19, 20, 21]. Additional criteria for the EARLY‐NEURO substudy include routine availability for collection and processing of biomarker samples, CT and EEG examinations. At participating sites, all patients unconscious, defined as unable to follow commands (FOUR‐M ≤ 3) at 24 h after cardiac arrest, will be included in the EARLY‐NEURO. Patients awake or deceased within 24 h after cardiac arrest will not undergo formal neuroprognostication. However, results of any prognostic examinations already performed and outcomes of these patients will be presented.

### 2.3 Exclusion Criteria

The STEPCARE‐trial exclusion criteria are non‐asphyxia related trauma or hemorrhage (including gastrointestinal bleeding) as the presumed cause of the arrest, suspected or confirmed intracranial hemorrhage, extracorporeal membrane oxygenation (ECMO) prior to randomization, pregnancy, and previous randomization to the STEPCARE trial [18, 19, 20]. Further exclusion criteria are inclusion in STEPCARE prior to the availability of biomarker collection kits. Patients not consenting or withdrawing consent to the study or with missing primary outcome will also be excluded.

### 2.4 Clinical Procedures

**FIGURE 2:**
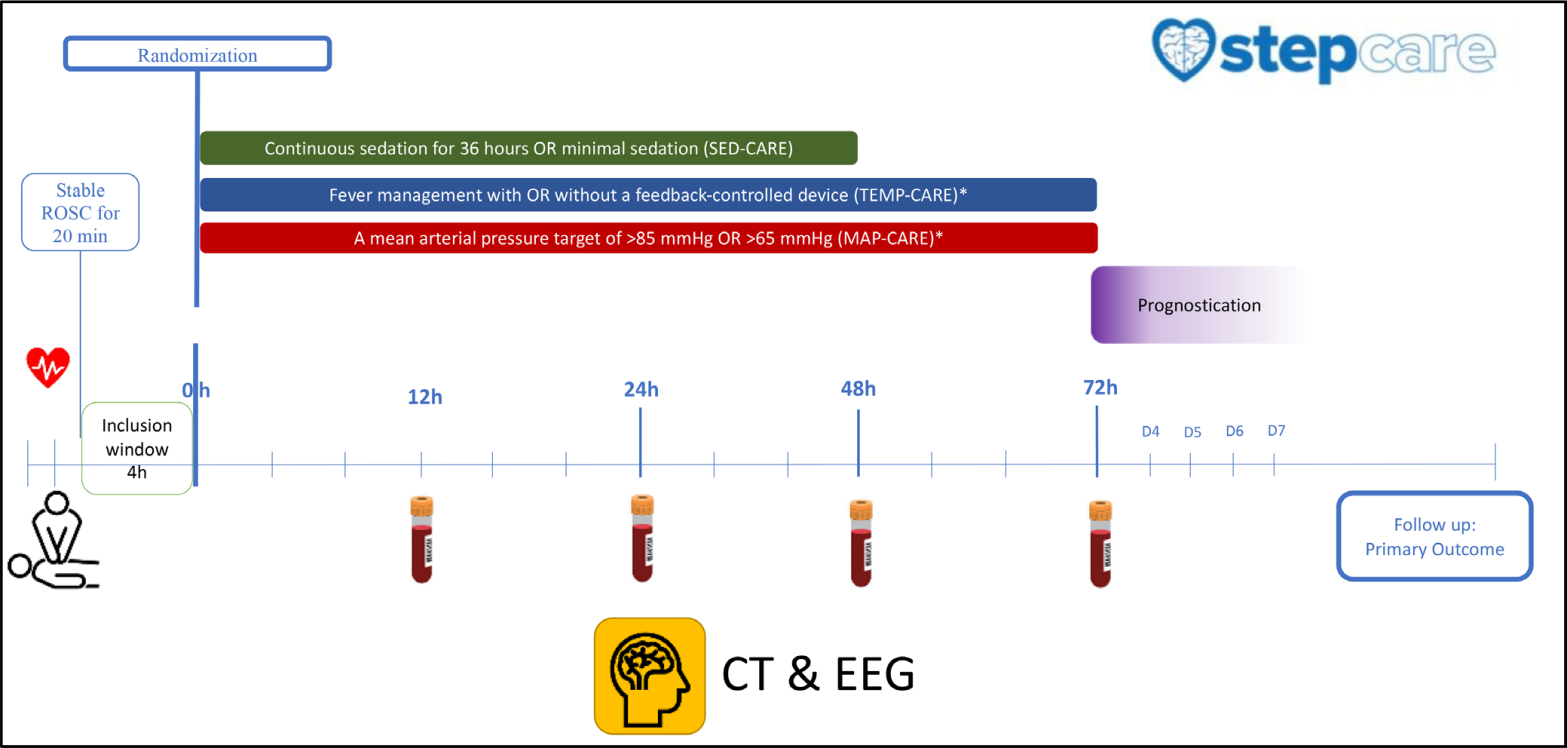
EARLY‐NEURO interventions and examinations. Overview of patient inclusion, interventions, and examinations of the STEPCARE and the timepoints for collection of biomarker samples, computed tomography (CT) and electroencephalogram (EEG) within the EARLY‐NEURO. Only NFL collected at 24 h will be included in the EARLY‐NEURO.

All STEPCARE patients are allocated to the treatment interventions for sedation, treatment of fever and mean arterial pressure as previously described (Figure 2) [18, 19, 20]. Briefly, the interventions are:

1. SED‐CARE: deep sedation targeting a Richmond Agitation and Sedation Scale (RASS) between −4 (unresponsive to voice) and −5 (unarousable) for 36 h versus minimal sedation as needed for clinical care [20].
2. TEMP‐CARE: fever management with or without a feedback‐controlled temperature control device [19]. In the intervention group, patients will have a device targeting a temperature of ≤ 37.5°C if temperature ≥ 37.8°C occurs within 72 h post‐randomization. All participants may receive standard fever treatment, including pharmacological agents according to local practice [19].
3. MAP‐CARE: mean arterial pressure target of either > 85 or > 65 mmHg. Adjustment of mean arterial pressure begins as soon as possible after randomization and continues for up to 72 h after randomization or until extubation, whichever occurs first [18].

During intensive care, all patients in the STEPCARE trial will be examined daily for the absence of pupillary and corneal reflexes, the presence of generalized status myoclonus or other clinical seizures, and the level of consciousness using the FOUR‐M score [23]. For sites participating in the EARLY‐NEURO, EEG and CT examinations and biomarker collections will be performed as outlined below.

The protocol for all STEPCARE patients is identical for trial interventions, criteria for withdrawal of life‐sustaining treatment and follow‐up, regardless of participation in the EARLY‐NEURO study.

### 2.5 Biomarkers

Serum and plasma samples will be collected at 12, 24, 48 and 72 h after admission to intensive care, processed on site and stored at −70 to −80°C before transport to a biobank. For the EARLY‐NEURO study, only levels of NFL at 24 h will be analysed by personnel blinded to clinical data after trial completion using a clinically available NFL assay [24]. Biomarker results from other timepoints will be reported separately. The NFL cutoffs for prediction of good outcome in the study will be defined based on the laboratory standards and established literature at the timepoint of reporting results. The authors plan to report results of normal NFL levels (primary analysis) and twice the normal NFL levels (exploratory analysis). The cut‐off level for poor outcome prediction at 24 h will be calculated based on the 98% specificity cut‐off from an ongoing individual patient data meta‐analysis including several large clinical out‐of‐hospital cardiac arrest trials. The cut‐off levels for NFL will be published before the availability of the NFL results from the EARLY‐NEURO. Another biomarker, Neuron‐specific enolase (NSE) is part of the ERC/ESICM algorithm for prognostication and may be analysed locally on site and used for neurological prognostication as part of clinical routine.

### 2.6 Electroencephalogram

Unconscious patients (FOUR‐M ≤3) may be examined with either continuous EEG‐monitoring spanning the time‐point 24 h after cardiac arrest or an intermittent routine‐EEG as early as possible ≥24 h post‐arrest. A reduced or full electrode montage may be used. According to the STEPCARE protocol, results of local EEG evaluations will not be blinded to the bedside physicians and may be used for neurological prognostication. Raw EEG data will be collected as European Data Format (EDF/EDF+) for standardized evaluation by a team of blinded experts. EEG patterns will be defined using the terminology of the ACNS [22]. Predicting a poor functional outcome: suppressed EEG background with amplitudes < 10 μV constituting > 50% of the recording (burst‐suppression) or the entirety of the recording (suppression) with or without superimposed discharges [7, 22], and predicting a good functional outcome: EEG background with < 10% suppression‐periods and amplitude > 20 μV (continuous or nearly continuous normal‐voltage background).

### 2.7 Computed Tomography

Within EARLY‐NEURO, a non‐contrast brain CT is part of routine examinations for unconscious patients as early as possible ≥ 24 h after cardiac arrest. Removal of EEG electrodes during CT examinations is recommended to reduce the risk of artefacts on images. CT data will be analyzed (1) within clinical routine by local radiologists not blinded to clinical data and (2) by a central blinded re‐evaluation according to a Standardized Operating Procedure as previously published [8, 25].

For the central blinded standardized re‐evaluation, original DICOM (Digital Imaging and Communications in Medicine) images of CTs acquired at 120 kV, preferably thin axial slices will be collected. The raters will be blinded to clinical data except for patient age as previously published [26]. Residual contrast from coronary angiography is not expected to affect outcome specificity but will be noted. The raters will evaluate CT images qualitatively, excluding images with artefacts that significantly affect interpretation. Further, the raters will answer whether the images fulfill the ERC/ESICM criteria of “diffuse and extensive hypoxic‐ischaemic brain injury” defined as an “extensive and bilateral loss‐ or reduction of gray‐white distinction in the basal ganglia and/or the frontoparietal cortex” [1, 25]. The Gray‐White Matter differentiation will be quantified by placing 8 Regions of Interest at the basal ganglia level (GWR‐8) as previously described, and the threshold of 1.10 for poor functional outcome will be validated [8, 27].

### 2.8 Neurological Prognostication

The STEPCARE trial will employ a conservative and strict protocol for prediction of a likely poor neurological outcome based on the 2021 ERC/ESICM recommendations (Table S1) [28]. Neuroprognostication < 72 h after randomization for the purpose of limiting life‐sustaining treatment is strongly discouraged as described in the Supporting Information. Patients awake and following commands (FOUR‐M 4) will not be subject to formal neuroprognostication.

### 2.9 Withdrawal of Life‐Sustaining Therapy

Decisions on withdrawal of life‐sustaining therapy (WLST) will be made by the treating physicians, when applicable together with the patient's relatives or legal representative, as required by local legislation. WLST based on a poor neurological prognosis only should not be performed prior to 72 h post‐arrest.

### 2.10 Outcomes

The primary outcome will be binary good or poor functional outcome at 6 months after cardiac arrest based on the modified Rankin Scale (mRS), evaluated by trained outcome assessors as described [29]. Poor outcome will be defined as mRS 4–6; moderately severe disability, severe disability, or death. In a sensitivity analysis, extremely poor outcome will be defined as mRS 5–6 (severe disability or death). The mRS is used to capture the overall impairment of physical and cognitive abilities in patients with neurological conditions and is recommended as a core outcome after cardiac arrest [30].

### 2.11 Data Collection and Management

Individual patient data regarding background characteristics, clinical features, and locally analyzed laboratory results will be prospectively collected from medical and ambulance service records. Detailed data including neurological status, body temperature, blood pressure values, and doses of vasoactive and sedative medications will be collected. Clinical data will be entered into a web‐based eCRF by site personnel. The software for the eCRF is provided by Spiral, New Zealand. Blood samples for the biobank will be collected as described above. EEG and CT raw data files will be collected pseudonymized and evaluated as described above.

### 2.12 Ethics, Information and Consent

The Swedish Ethical review board approved the STEPCARE trial including the EARLY‐NEURO on 18th June 2022 (Dnr. 2022‐02425) and amendments including the biomarker substudy (Dnr. 2023‐00198‐02 and Dnr. 2025‐01116‐02). Ethics committees in each participating country have approved the protocol and decided whether initial written informed consent will be waived, deferred, or obtained from a legal surrogate. In addition, informed consent will be obtained from each patient who regains mental capacity.

### 2.13 Statistical Analyses

**FIGURE 3:**
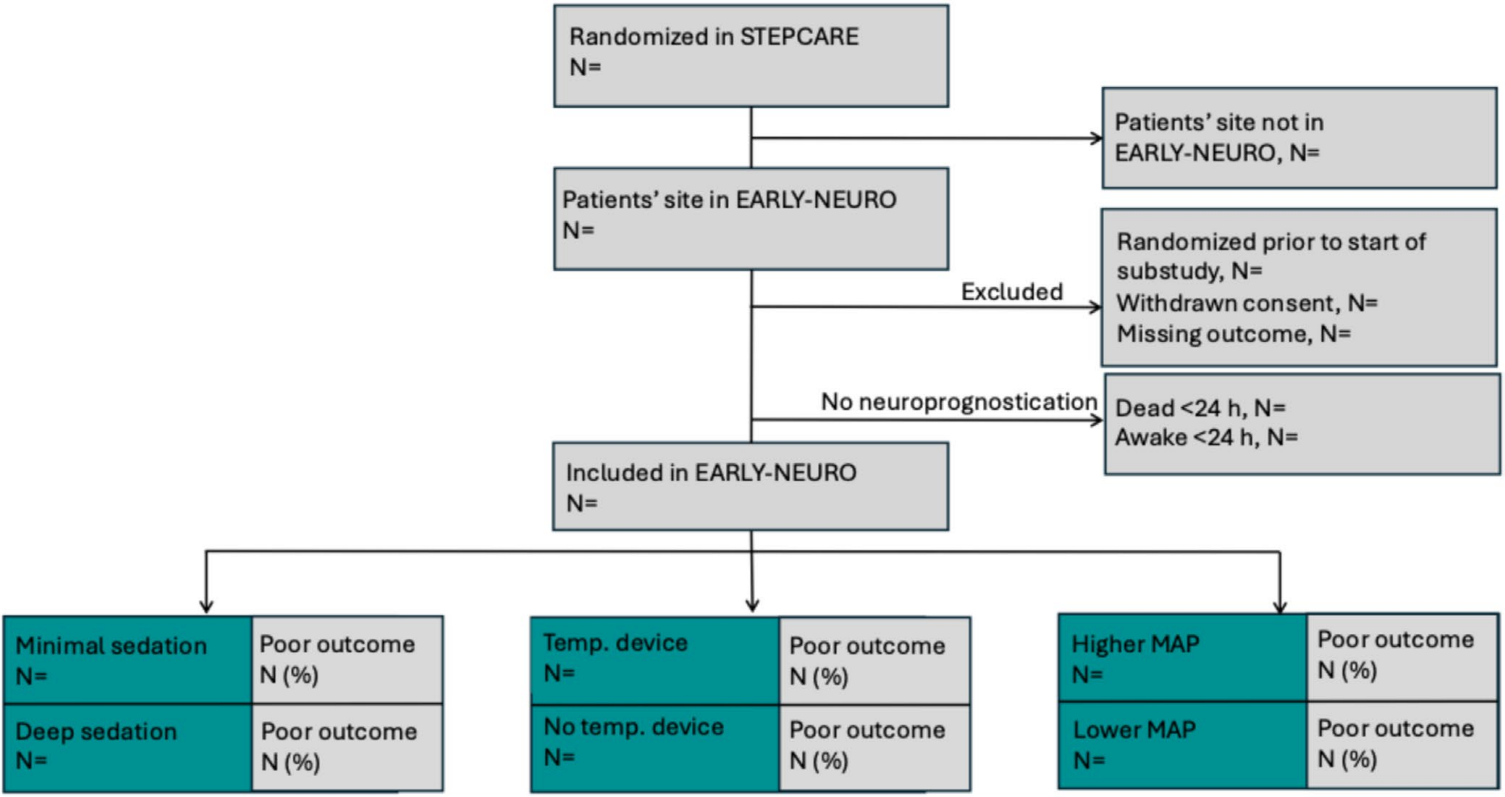
Flowchart of patient inclusion. Unconsciousness is defined as Full Outline of Unresponsiveness Motor score (FOUR‐M) ≤ 3. *N* = numbers. MAP = mean arterial pressure. We will present data for patients at participating sites who are unconscious at 24 h after admission to intensive care, and for patients who are not undergoing neuroprognostication since they are already awake or deceased prior to prognostication.

Results will be reported according to the Standards for Reporting Diagnostic accuracy studies (STARD) [31]. Reasons for exclusion will be presented according to the CONSORT (Consolidated Standards of Reporting Trial) flowchart as described (Figure 3). Patient characteristics will be described as in Table 1. We will further describe eligible patients not undergoing neuroprognostication (awake or deceased prior to 24 h after cardiac arrest).

The main results will be the prognostic performance of the three diagnostic methods NFL, EEG and CT for prediction of binary functional outcome (good versus poor) at 6 months. We will present sensitivity, specificity, positive predictive value and negative predictive value with 95% confidence intervals for poor outcome prediction for each prognostic method, and for any two combinations of pathological NFL, CT and EEG. The prognostic performances will be presented as pooled analysis of all data available, and separately for each intervention group within the STEPCARE trial as specified above. Based on our previous publications, an NFL cutoff at 2% FPR at 24 h yielded sensitivities between 60% and 69%, with 95% confidence intervals for the 0.98 specificity of 0.95–1.00 and 0.94–1.00, respectively [11, 17]. We expect that the number of included patients in the EARLY‐NEURO will be of similar sample size, or larger than these previously published studies, thus with sufficient statistical power to answer our primary research questions.

**TABLE 1:** Patient characteristics.

	Included (N=)^c^	Excluded (N=)^c^
Demographic characteristics		
Age—year	x	x
Male sex—no. (%)	x	x
Female sex—no. (%)	x	x
Medical history—no. (%)		
Estimated pre‐arrest functional status		
Independent in basic activities of life	x	x
Dependent in basic activities of life	x	x
Percutaneous coronary intervention	x	x
Coronary artery bypass grafting	x	x
Heart failure with pharmacologic treatment	x	x
Implantable cardioverter defibrillator	x	x
Hypertension with pharmacologic treatment	x	x
Stroke or transitory ischemic attack	x	x
Chronic obstructive pulmonary disease	x	x
Diabetes mellitus	x	x
Kidney disease^a^	x	x
Characteristics of cardiac arrest—no. (%)		
Scene of cardiac arrest		
Home	x	x
Public place	x	x
Other	x	x
Bystander witnessed cardiac arrest	x	x
Bystander performed cardiopulmonary resuscitation	x	x
Changing rhythms (any 2 of VF/PEA/asystole)	x	x
Adrenaline administered^b^	x	x
First monitored rhythm—no. (%)		
Ventricular fibrillation	x	x
Ventricular tachycardia	x	x
ROSC after bystander‐initiated defibrillation	x	x
Unknown shockable rhythm	x	x
Pulseless electrical activity	x	x
Asystole	x	x
Unknown non‐shockable rhythm	x	x
Minutes from cardiac arrest to ROSC—min (IQR)	x	x
Clinical characteristics at hospital admission:		
First recorded temperature—(°C)	x	x
First pH	x	x
Clinical characteristics at admission to intensive care		
Circulatory shock^d^—no. (%)	x	x
ST‐segment elevation myocardial infarction—no. (%)	x	x
Clinical characteristics during intensive care		
Time to awakening^e^—hours (IQR)	x	x
Randomization		
Minimal sedation‐no. (%)	x	x
Deep sedation‐no. (%)	x	x
Temperature management with a device‐no. (%)	x	x
Temperature management without a device‐no. (%)	x	x
Mean Arterial Pressure target > 85 mmHg‐no. (%)	x	x
Mean Arterial Pressure target > 65 mmHg‐no. (%)	x	x
Neurological prognostication		
FOUR‐M—score at 24 h after cardiac arrest (IQR)	x	x
FOUR‐M—score 0, no response—no. (%)	x	x
FOUR‐M—score 1, extension posturing‐no. (%)	x	x
FOUR‐M—score 2, flexion posturing‐no. (%)	x	x
FOUR‐M—score 3, localizing pain‐no. (%)	x	x
FOUR‐M—score 4, awake and following commands‐no. (%)	x	x
Corneal reflexes bilaterally absent‐no. (%)	x	x
Pupillary reflexes bilaterally absent‐no. (%)	x	x
Early status myoclonus present‐no. (%)	x	x
SSEP N20 amplitudes bilaterally absent‐no. (%)	x	x
NSE elevated at 48 and/or 72 h‐no. (%)	x	x
MRI diffuse and extensive HIE‐no. (%)	x	x
EEG performed—no.(%)	x	x
Time to EEG—hours (IQR)	x	x
Full montage EEG performed‐no (%)	x	x
Simplified continuous EEG performed‐no (%)	x	x
CT performed—no. (%)	x	x
Time to CT—hours (IQR)	x	x
NFL results at 24 h available—no. (%)	x	x
Neuroprognostication performed—no. (%)	x	x
Time from cardiac arrest to neuroprognostication—hours (IQR)	x	x
Poor outcome likely according to STEPCARE criteria—no. (%)	x	x
Outcome at 6 months follow‐up, modified Rankin Scale (mRS)		
Binary good outcome, mRS 0–3—no. (%)	x	x
Binary poor outcome, mRS 4–6—no. (%)	x	x
mRS 0—no. (%)	x	x
mRS 1—no. (%)	x	x
mRS 2—no. (%)	x	x
mRS 3—no. (%)	x	x
mRS 4—no. (%)	x	x
mRS 5—no. (%)	x	x
mRS 6—no. (%)	x	x
Withdrawal of life‐sustaining therapy (WLST)		
WLST, any reason—no. (%)	x	x
Time to WLST, any reason—hours (IQR)	x	x
WLST, neurological reason only—no. (%)	x	x
Time to WLST, neurological reason only—hours (IQR)	x	x

Abbreviations: EEG = electroencephalogram; IQR = interquartile range; MRI = brain magnetic resonance imaging with diffuse and extensive hypoxic‐ischaemic brain injury according to evaluation by local radiologist; NSE = blood levels of NSE above 60 ng/mL at 48 and/or 72 h according analysis per clinical routine; PEA = pulseless electrical activity; ROSC = return of spontaneous circulation; SSEP N20 = bilaterally absent N20 amplitudes on somatosensory evoked potentials according to evaluation by local expert; VF = ventricular fibrillation.

^a^ Estimated glomerular filtration rate < 30 mL/min/1.73 m^2^.

^b^ Prior ROSC and hospital arrival by emergency medical service.

^c^ Full Outline of Unresponsiveness (FOUR) motor scores range from 0 to 4, with higher scores indicating better motor function.

^d^ Shock on admission is defined as a systolic blood pressure of less than 90 mmHg for at least 30 min or the need for supportive measures to maintain a systolic blood pressure ≥ 90 and end organ hypoperfusion (cool extremities or urine output of less than 30 mL/h and heart rate > 60/min).

^e^ Awakening defined as the patient was awake and obeying verbal commands for any reason during their ICU stay.

We will present sensitivity, specificity, positive predictive value and negative predictive value with 95% confidence intervals for the prediction of good outcome for each prognostic method alone and for combinations of normal NFL, CT, EEG and FOUR‐M score 3 (localizing pain) (Tables 2 and 3).The functional outcome and the fulfilled number of criteria for prediction of good/poor outcome will be presented for included patients with FOUR‐M ≤ 3 (Table S2). We will also present available data for patients not subject to neuroprognostication since they were already awake, or dead within 24 h.

**TABLE 2:** Prognostic performance for poor functional outcome.

	Criteria of poor outcome	Specificity (95% CI)	Sensitivity (95% CI)	PPV (95% CI)	NPV (95%)	TP	FP	TN	FN	N=
Single	EEG highly malignant									
	CT diffuse and extensive HIE									
	High NFL									
Combined	EEG and CT									
	EEG and NFL									
	CT and NFL									
	EEG, CT and NFL									

*Note:* We will present prognostic performances for each poor outcome criteria separately, and in combination with the other prognostic methods. Functional outcome will be dichotomized into good and poor according to the modified Rankin Scale (0–3 versus 4–6) at six months. PPV; positive predictive value. NPV; negative predictive value. TP; true positive (pathological finding and poor functional outcome). FP; false pathological (pathological finding and good functional outcome). TN; true negative (non‐pathological finding and good functional outcome). FN; false negative (non‐pathological findings and poor functional outcome). Results will be presented as numbers or percentages with 95% confidence intervals.

**TABLE 3:** Prognostic performance for good functional outcome.

	Criteria of good outcome	Sensitivity (95% CI)	Specificity (95% CI)	NPV (95% CI)	PPV (95%)	TN	FN	TP	FP	N=
Single	FOUR M 3 localizing pain									
	EEG cont. normal voltage									
	CT without diffuse and extensive HIE									
	Normal NFL									
Combined	FOUR M 3 and EEG									
	FOUR M 3 and CT									
	FOUR M 3 and NFL									
	EEG and CT									
	EEG and NFL									
	CT and NFL									
	FOUR M3, EEG, CT and NFL									

*Note:* We will present prognostic performances for each good outcome criteria separately, and in combination with the other prognostic methods. Functional outcome will be dichotomized into good and poor according to the modified Rankin Scale (0–3 versus 4–6) at 6 months. PPV; positive predictive value. NPV; negative predictive value. TP; true postitive (non‐normal finding and poor functional outcome). FP; false pathological (non‐normal finding and good functional outcome). TN; true negative (normal finding and good functional outcome). FN; false negative (normal finding and poor functional outcome). Results will be presented as numbers or percentages with 95% confidence intervals.

For the main results, we will perform sensitivity analyses including alternative definition of good (mRS 0–4) and poor (mRS 5–6) functional outcomes. This alternative threshold aims to test the robustness of the prognostic markers in predicting and limiting treatment in the most severe outcomes only (severe disability or death).

Secondary results include the prognostic performance of all prognostic methods as specified above, presented by intervention group (sedation, temperature management and mean arterial pressure).

Pre‐defined tertiary analyses listed in the hypotheses section will be presented in subsequent manuscripts.

### 2.14 Trial Status and Timeline

Randomization began in August 2023 and trial sites have been added gradually. The last patient included in the STEPCARE is expected in the middle of 2026, with the last 6‐month follow‐ups estimated to be finished by the end of 2026. Results from the EARLY‐NEURO will be presented after the results of the trial interventions have been published [18, 19, 20, 21].

## 3 Discussion

Nested within an international randomized clinical trial, the EARLY‐NEURO study is uniquely positioned to address critical knowledge gaps in post‐cardiac arrest care. Primarily, it investigates whether neurological prognostication can be reliably performed ahead of current guideline‐recommended timelines. Furthermore, the study examines the differential predictive accuracy of various diagnostic modalities for both favorable and unfavorable outcomes, while specifically evaluating the confounding influence of sedation on biomarkers, neuroimaging, and neurophysiology.

The conservative approach of the ERC/ESICM guidelines has been crucial to postpone neuroprognostication with the aim of reducing the confounding effects of sedative drugs on wakefulness and to allow time for patients with good prognosis to have a spontaneous recovery. During recent years, methods to asses the degree of brain injury while the patient is still sedated have been advanced including quantification of gray‐white matter differention on brain CT and NFL in blood. Further, sedation in commonly used doses in the post cardiac arrest setting unlikely affects poor outcome prediction with EEG, but robust evidence is lacking [7, 32]. These three methods all perform well prognostically around 24 h post arrest. With a combination of such reliable test results, potential effects of sedation on the level of consciousness may be less problematic.

In addition, the allocation of patients to minimal and deep sedation targets in STEPCARE gives us a unique opportunity to study whether sedation levels influence the reliability of the investigated methods and their combinations.

If our primary hypotheses prove correct, this will pave the way for modification of the current ERC/ESICM algorithm with earlier identification of the most severely brain injured patients and those with no or minimal injury.

The target methods for prognostication in EARLY‐NEURO, brain‐CT, EEG and NFL, were selected because of the general availability of CT and EEG, and the likely broad implementation of NFL which is now available on standard diagnostic instruments from several manufacturers.

Absent N20 median nerve somatosensory evoked potentials (SSEP) are highly specific for poor outcome prediction [33]. Nonetheless, SSEP is not widely available and has traditionally not been considered for good outcome prediction. Clinical examinations of bilaterally absent corneal‐ and pupillary reflexes, or early status myoclonus are also highly specific for poor outcome prediction, but compared to CT, EEG or NFL, their sensitivity seems limited for good outcome prediction [3, 5, 11, 34, 35]. EEG and CT represent the most commonly used add‐on tools to neurological prognostication and both can also be evaluated by experts who work remotely [9]. Thus, together with NFL, which is already becoming available as a routine assay, a standardized multimodal neuroprognostication with CT and EEG should be available even in hospitals that are not specialized cardiac arrest centers.

The 2025 ERC/ESICM guideline algorithm now includes a fourth step evaluating criteria for good neurological prognosis [1]. In a multicenter study matching patients from countries where treatment limitations are commonly performed with patients not subjected to treatment limitations, we found that patients not fulfilling ERC/ESICM criteria of a likely poor outcome are at risk of treatment withdrawal despite a potentially good outcome [2]. Thus, the inclusion of favorable predictors in the algorithm is of utmost importance to decrease uncertainty of neuroprognostication to guide treatment decisions [3].

EEG, NFL, and localizing pain upon stimulus (FOUR‐M score 3) have demonstrated their ability to identify patients with a good neurological prognosis within the first 24 h after cardiac arrest [5, 36, 37]. A normal magnetic resonance imaging of the brain at 2–5 days post‐arrest is another predictor of favorable outcome [4]. We presume that the absence of diffuse and extensive HIE on non‐contrast CT may not be the ideal predictor of good neurological outcome, since the severity of HIE increases within the first days after cardiac arrest [8, 38]. Due to the availability of CT, we will nonetheless evaluate whether a standardized qualitative and quantitative CT assessment can be useful for prediction of good outcome.

In summary, the EARLY‐NEURO study seeks to expedite the prognostic timeline and enable earlier identification of recovery potential. By integrating clinical assessments of consciousness with neuroimaging, neurophysiology, and a brain injury biomarker, the study aims to reduce prognostic uncertainty well before current standard‐of‐care windows.

### 3.1 Strengths and Limitations

Strengths of this study include the prospective international design, collection of blood biomarkers, a standardized approach to intensive care treatment including sedation, temperature, blood pressure treatment, prediction of neurological outcome, withdrawal of life‐sustaining therapy, and structural blinded follow‐up at 6 months. The standardized evaluation of CT and EEG by raters blinded to clinical information, together with quantitative NFL levels, is designed to reduce interrater variability in neuroprognostication. Withdrawal of therapy prior to 72 h after randomization based on a presumed futile neurological prognosis is strongly discouraged in STEPCARE.

Limitations of the EARLY‐NEURO include the availability of on‐site evaluations of CT, EEG, and biomarkers for the treating physicians as part of clinical routine. Information from prognostic methods will overlap with the blinded re‐evaluations in this study, and the risk of a self‐fulfilling prophecy can therefore not be excluded. To address this, we will use levels of NFL as a surrogate marker of brain injury. Further, both functional outcome and death may be influenced by non‐neurological co‐morbidities.

## 4 Conclusions

An accelerated, multimodal prognostic strategy has the potential to protect patients with favorable recovery prospects and avoid unnecessary treatment for patients with a definite poor prognosis. This study will determine the diagnostic yield of such an approach and investigate whether sedation, blood pressure, or temperature management confound prognostication.

## Author Contributions

M. Moseby‐Knappe and T. Cronberg drafted the manuscript. All authors contributed to the study design, critically revised and approved the final version of the manuscript.

## Funding

The STEPCARE trial is funded by The Swedish Research Council, The Swedish Heart‐lung Foundation, ALF‐project funding within the Swedish Health Care, The Academy of Finland, Sigrid Jusélius Foundation (Finland), Hospital District of Helsinki and Uusimaa (Finland), Medicinska Understödsföreningen Liv och Hälsa (Sweden), Medical Research Future Fund (Australia), Health Research Council of New Zealand, the Clinical Research Programme Directorate of Health Ministry of Health and Social Security (Luxembourg), the Regional Research Council of Region Halland, and the Fondation Cœur‐Daniel Wagner under the aegis of the Fondation de Luxembourg (Luxembourg). The STEPCARE EARLY‐NEURO study is further funded by regional research funds in the Scania region, and the Skane University Hospital Foundations.

## Conflicts of Interest

Markus Skrifvars received a speakers fee from BARD Medical (Ireland) 2022 and is a member of the editorial board of Acta Anaesthesiologica Scandinavica. Christoph Leithner has received research support from the Laerdal Foundation. Jens Nee reports receiving lecture fees from BD and Becton Dickinson GmbH. No further conflicts of interest were reported.

## Supporting information


**Table S1:** Criteria for a likely poor neurological outcome in STEPCARE.
**Table S2:** Concordance of predictors of good and poor functional outcome according to the primary EARLY‐NEURO hypotheses.

## Data Availability

Data sharing not applicable to this article as no datasets were generated or analysed during the current study.
