# Supplementary material for: Early Prediction of Neurological Outcome After Cardiac Arrest‐Rationale and Design of the Prospective International Observational EARLY‐NEURO, a STEPCARE Substudy
Source: Acta Anaesthesiol Scand. 2026 Apr 17;70(5):e70239. doi: 10.1111/aas.70239 (PMC13088753; doi:10.1111/aas.70239)
Supplement: Supplementary file 1 — Table S1: Criteria for a likely poor neurological outcome in STEPCARE. Table S2: Concordance of predictors of good and poor functional outcome according to the primary EARLY‐NEURO hypotheses. [file AAS-70-0-s001.docx]

**Early prediction of neurological outcome after cardiac arrest**

**- Rationale and design of the prospective international observational EARLY-NEURO, a STEPCARE substudy**

**Supplementary Appendix**

**Supplementary methods**

Neurological prognostication

The STEPCARE trial will employ a conservative and strict protocol for neurological prognostication based on the 2021 ERC/ESICM recommendations. The physician performing the prognostication will be a neurologist, intensivist or other specialist experienced in neuroprognostication who has not been involved in patient care. The prognosticator should be blinded for group allocations and the findings of the centralized evaluations of CT, EEG and biomarkers, but not for relevant clinical data. Prognostication will be performed on all participants who have no response, a stereotypic extensor response or a stereotypic flexor response to bilateral central and peripheral painful stimulation at ≥ 72 h after randomization, and who are still receiving intensive care at 72 hours after randomisation. Neuroprognostication <72 hours after randomisation is strongly discouraged.

Prior to neuroprognostication, confounding factors such as residual effects of sedation or metabolic derangements must be excluded as per ERC/ESICM recommendations. The clinical examination used for prognostication should not be performed earlier than 72 hours after randomisation but may be delayed due to lingering sedation or for practical reasons (such as weekends or national holiday). Results from additional examinations performed <72 hours may be included in the assessment if performed according to ERC/ESICM recommendations. The STEPCARE criteria of a likely poor outcome is a conservative adaptation of the 2021 ERC/ESICM recommendations (eFig. 2). The result of the prognostication will be categorised as “YES” or “NO”, based on the answer to the question “Does this patient fulfill the STEPCARE criteria for a likely poor neurological outcome?” using the trial checklist provided (eTable 1). This assessment will be documented in the case report form and will be communicated to the treating clinician. Results of neurological prognostication and the potential decision to withdraw active intensive care are closely related but will be considered separate entities.

**eTable 1. Criteria for a likely poor neurological outcome in STEPCARE**

|  |
| --- |
| **In the STEPCARE trial prognosis is considered *likely poor* if criteria A, B and C are all fulfilled:** |
| **A.** Confounding factors such as severe metabolic derangement and lingering sedation have been ruled out. The ERC/ESICM recommends awaiting 5 half-lives of the sedative with the longest half-life prior to clinical evaluation. |
| **B.** The patient has no response, a stereotypic extensor response or a stereotypic flexor response to bilateral central and peripheral painful stimulation at ≥ 72 h after randomization (FOUR M$\leq$ 2). |
| **C.** At least two of the below mentioned signs of a poor prognosis are present:  **C1.** No pupillary AND corneal reflexes ≥72 hours after randomization  **C2.** Bilaterally absent SSEP N20-potentials  **C3.** Early generalized and persisting myoclonus (myoclonic jerks persisting ≥30 min) ≤72 hours after randomization  **C4.** Highly malignant and unreactive EEG-pattern >24 hours after randomization  **C5.** Diffuse and extensive hypoxic brain injury on CT/MRI  **C6.** High NSE >60 ug/L at 48 and/or 72 hours after randomization |

The result of the prognostication will be categorized as “YES” or “NO”, based on the answer to the question *“Does this patient fulfil the STEPCARE criteria for a likely poor neurological outcome?”*.

**eTable 2. Concordance of predictors of good and poor functional outcome according to the primary EARLY-NEURO hypotheses**

|  | **Number of poor outcome predictors**  Poor outcome likely $\geq$2 pathological predictors | | | | |
| --- | --- | --- | --- | --- | --- |
| **Number of good outcome predictors**  Good outcome likely $\geq$2 predictors |  | **0** | **1** | **2** | **3** |
|  | **0** | Good: N (%)  Poor: N (%) | Good: N (%)  Poor: N (%) | Good: N (%)  Poor: N (%) | Good: N (%)  Poor: N (%) |
|  | **1** | Good: N (%)  Poor: N (%) | Good: N (%)  Poor: N (%) | Good: N (%)  Poor: N (%) | Good: N (%)  Poor: N (%) |
|  | **2** | Good: N (%)  Poor: N (%) | Good: N (%)  Poor: N (%) | Good: N (%)  Poor: N (%) | Good: N (%)  Poor: N (%) |
|  | **3** | Good: N (%)  Poor: N (%) | Good: N (%)  Poor: N (%) | Good: N (%)  Poor: N (%) | Good: N (%)  Poor: N (%) |
|  | **4** | Good: N (%)  Poor: N (%) | Good: N (%)  Poor: N (%) | Good: N (%)  Poor: N (%) | Good: N (%)  Poor: N (%) |

This table will include all prognostic examinations of good and poor outcome in patients with FOUR-M $\leq$3 at 24 hours after cardiac arrest. We will present numbers (N) and percentages of patients with good and poor functional outcome (mRS 0-3 versus 4-6 at six months’ follow-up). We will present a similar table for patients awake and obeying commands, or dead prior to 24 hours after cardiac arrest.
